# Genetics and Mitochondrial Abnormalities in Autism Spectrum Disorders: A Review

**DOI:** 10.2174/138920211796429745

**Published:** 2011-08

**Authors:** Sukhbir Dhillon, Jessica A Hellings, Merlin G Butler

**Affiliations:** Departments of Psychiatry & Behavioral Sciences and Pediatrics, Kansas University Medical Center, Kansas City, Kansas 66160, USA

**Keywords:** Autism spectrum disorders (ASD), mitochondrial DNA (mtDNA) mutations and depletion, oxidative stress, nuclear genes, lactate/pyruvate ratios, genetic causation.

## Abstract

We review the current status of the role and function of the mitochondrial DNA (mtDNA) in the etiology of autism spectrum disorders (ASD) and the interaction of nuclear and mitochondrial genes. High lactate levels reported in about one in five children with ASD may indicate involvement of the mitochondria in energy metabolism and brain development. Mitochondrial disturbances include depletion, decreased quantity or mutations of mtDNA producing defects in biochemical reactions within the mitochondria. A subset of individuals with ASD manifests copy number variation or small DNA deletions/duplications, but fewer than 20 percent are diagnosed with a single gene condition such as fragile X syndrome. The remaining individuals with ASD have chromosomal abnormalities (e.g., 15q11-q13 duplications), other genetic or multigenic causes or epigenetic defects. Next generation DNA sequencing techniques will enable better characterization of genetic and molecular anomalies in ASD, including defects in the mitochondrial genome particularly in younger children.

Leo Kanner described autism in 1943 in 11 children manifesting withdrawal from human contact as early as age 1 year postulating origins in prenatal life [1]. The Diagnostic and Statistical Manual of Mental Disorders IV-Text Revised (DSM-IV-TR) described autism as a complex neurobehavioral disorder characterized by deficiencies in social interaction, impaired communication skills and repetitive stereotypic behavior with onset prior to 3 years of age [2]. Several conditions including full-syndrome autism (Autistic Disorder), Asperger Syndrome and Pervasive Developmental Disorder Not Otherwise Specified (PDD-NOS) are now grouped as Autism Spectrum Disorders (ASD) also known as Pervasive Developmental Disorders (PDDs) [2-4]. 

Symptoms of ASD usually begin in early childhood with evidence of delayed development before age 3 years. The American Academy of Pediatrics recommends autism screening for early identification and intervention by at least age 12 months and again at 24 months. Rating scales helpful in establishing the diagnosis are Autism Diagnostic Interview- Revised (ADI-R) and the Autism Diagnostic Observation Schedule (ADOS) in combination with clinical presentation [5, 6]. 

## GENETIC CONTRIBUTIONS TO AUTISM SPECTRUM DISORDERS

The etiology of ASD is complex and encompasses the roles of genes, the environment (epigenetics) and the mitochondria. Mitochondria are cellular organelles that function to control energy production necessary for brain development and activity. Researchers are increasingly identifying mitochondrial abnormalities in young children with ASD since the most severe cases present early with features of ASD. Better awareness and more accurate and detailed genetic and biochemical testing are now available for the younger patient presenting with developmental delay or behavioral problems. 

Epidemiologic and family studies suggest that genetic risk factors are present. Monogenic causes are identifiable in less than 20 percent of subjects with ASD. The remaining subjects have other genetic or multigenic causes and/or epigenetic influences which are environmental factors altering gene expression without changing the DNA sequence [7-10]. Epigenetic factors in ASD have been reviewed by Grafodatskaya *et al.* [11]. The recurrence risk for ASD varies by gender for the second child to be affected (4% if the first child affected is female and 7% if a male). The recurrence rate increases to 25-30% if the second child is also diagnosed with ASD. Studies have shown that among identical twins, if one child has ASD, then the other has a 60 to 95% chance of being affected. 

Fragile X syndrome and tuberous sclerosis are the most common single gene conditions associated with ASD. The commonest chromosomal abnormality in non-syndromal autism is duplication of the 15q11-q13 region, accounting for 5% of patients with autism. Large microdeletions in chromosome 16p11.2 and 22q regions account for another 1% of cases [12]. The rapid rise in the incidence of ASD in the past 30 years, apart from improved identification, points to environmental factors acting on essentially unchanged genetic predispositions involving nuclear and mitochondrial DNA since *de novo* changes in genes are unlikely to occur so quickly. Specific genetic and cytogenetic conditions associated with ASD are summarized in a recent review [13].

The role and importance of genetic testing for individuals with ASD is well recognized [14] with various studies showing yields of 6% to 40% with newer testing methods [15, 16]. Early studies by Miles and Hillman [15] tested 94 children clinically diagnosed with ASD and found that 6 of 94 (6%) had identifiable genetic disorders. Herman *et al.* [17] later found genetic causes in 7 of 71 (10%) subjects with ASD. Schaefer and Lutz [16] used a three- tier clinical genetic approach to identify causes in 32 clinically diagnosed children with ASD, and reported positive genetic findings in 13 subjects (40%). These included 5% with a high resolution chromosomal abnormality, 5% with fragile X syndrome, 5% with Rett syndrome (*MECP2* gene defects in females), 3% with *PTEN* gene mutations in those with a head circumference > 2.5 SD, approximately 10% with other genetic syndromes (e.g., tuberous sclerosis) and 10% with small deletions or duplications identified using chromosomal microarrays.

High resolution chromosome analysis detects 3 to 5 megabase-sized abnormalities; however, new technology using DNA or chromosomal microarrays can identify abnormalities 100 times smaller. Therefore, microdeletions and duplications may now be identified with microarrays in individuals with ASD who previously had normal cytogenetic testing. Children with ASD show a higher prevalence of microdeletions and duplications, particularly involving chromosome regions 1q24.2, 3p26.2, 4q34.2, 6q24.3 and 7q35 including those with non-syndromal ASD [18]. Therefore, if cytogenetic analysis is negative in clinically diagnosed ASD, testing for microdeletions and duplications with newer techniques is warranted. 

Shen and coworkers [19] studied genetic testing results from a cohort of 933 patients with ASD including G-banded karyotypes, fragile X testing and chromosomal microarrays. They reported abnormal karyotypes in 19 of 852 patients (2.2.%), abnormal fragile X testing in 4 of 861 patients (0.5%) and microarray identified deletions or duplications in 154 of 848 patients (18.2%). Fifty-nine of 154 subjects (38.3%) were associated with known genomic disorder variants with possible significance to ASD. With the exception of recurrent deletions or duplications of chromosome 16p11.2 [20] and chromosome 15q13.2q13.3 [21], most copy number changes were unique. 

Wang *et al.* [22] reported the results from a genome-wide association study of 3,101 subjects representing 780 families with affected children and a second cohort of 1,204 affected subjects along with 6,491 controls. All of their subjects were of European ancestry. They found a strong association with six nucleotide polymorphisms between cadherin 10 (*CDH10*) and cadherin 9 (*CDH9*) genes. The latter two genes encode neuronal cell-adhesion molecules. These findings were replicated in two independent cohorts and demonstrate an association with susceptibility to ASD. 

Sebat *et al.* [23] studied 165 individuals with autism grouped into 118 simplex families without a family history of autism and 47 multiplex families with multiple affected siblings and compared them with control groups without autism. They reported that 10.2% (12 of 118) of simplex families showed copy number variants (CNVs), 2.6% (2 of 77) with CNVs in individuals with autism from multiplex families and 1% (2 of 196) with CNVs among normally developing children. The majority of CNVs were of the deletion type. Thus, CNVs were significantly more common in the sporadic form of autism than in those with a family history due to single gene mutations not detectable by DNA deletion or duplication analysis.

As a result of research into nucleotide sequences, microdeletions and duplications in children with ASD can now be identified including syntaxin binding protein 5 (*STXBP5*) and neuronal leucine rich repeat 1 (*NLRR1*) genes. Syntaxin 5 regulates synaptic transmission at the presynaptic cleft and is known to inhibit synapse formation. Syntaxin 1 protein is increased in those with high functioning autism. The role of *NLRR1* at the synaptic level is unknown but is thought to be related to neuronal growth [24]. 

An autism genome-wide copy number variation study reported by Glessner *et al.* [25] in a large cohort of ASD cases compared with controls showed that *NRXN1 *and *CNTN4* genes play a role. They also described new susceptibility genes, *NLGN1* and *ASTN2* which encode neuronal cell-adhesion molecules and other genes involved in the ubiquitin pathways (*UBE3A, PARK2, RFWD2* and *FBXO40*), two important gene networks expressed in the central nervous system.

Next generation DNA sequencing is currently underway, allowing for rapid and efficient detection of mutations at the nuclear and mitochondrial DNA (mtDNA) level in human investigations and becoming part of clinical workup. Heteroplasmy or the existence of multiple mtDNA types within cells of an individual, is detectable using standard molecular genetic techniques which focus on hypervariable regions of the mitochondrial genome. With high-throughput next generation sequencing of the complete human mtDNA, which is faster and more powerful, accurate detection of heteroplasmy can be made throughout the mitochondrial genome not just in the hypervariable regions located in the cytoplasm of the cell enabling the study of correlation with diseases. 

Recently, Li *et al.* [26] sequenced the mitochondrial genome of 131 healthy individuals of European ancestry. They identified 37 heteroplasmies at 10% frequency or higher in 32 individuals and located at 34 different sites of the mtDNA indicating that variation commonly occurs in mtDNA. These variations may impact on energy levels and influence brain development and function. Next generation sequencing should provide novel insights into genome-wide aspects of mtDNA variation or heteroplasmy useful in the study of human disorders including autism. 

With a significant percentage of children with autism presenting with metabolic abnormalities (e.g., high lactate levels) and other biochemical disturbances, identification of mitochondrial disorders is critical for early treatment. Long standing mitochondrial dysfunction can lead to major health complications and damage. If identified early, mitochondrial disorders can be managed with improved longevity and quality of life. Medical intervention and therapies are now available to specifically target the biochemical defect in the mitochondria and to improve function and bioenergy utilization and diminish the neurological insults that would occur if left untreated. 

## MITOCHONDRIAL ROLE IN AUTISM

### Metabolic Effects

Inborn errors of metabolism may contribute to at least 5% of cases with ASD [27]. Deficiency of certain enzymes in metabolic disorders leads to an accumulation of substances that can cause toxic effects on the developing brain, contributing to ASD. A common example is phenylketonuria, an autosomal recessive condition leading to excessive phenylalanine levels, mental retardation and ASD, if not diet-controlled. Other related conditions include purine metabolism errors (adenylosuccinase deficiency and adenosine deaminase deficiency), creatine deficiency syndromes, Smith-Lemli-Opitz syndrome, biotinidase deficiency and histidinemia. Early screening and treatment of these conditions may have a positive impact in preventing disease progress. For example, the prevalence of individuals affected with phenylketonuria has decreased from 20% to 6% with early detection during infancy and dietary intervention to control phenylalanine intake [28, 29]. Once mitochondrial abnormalities are better elucidated, similarly preventive intervention may become available.

**Fig. (1).**
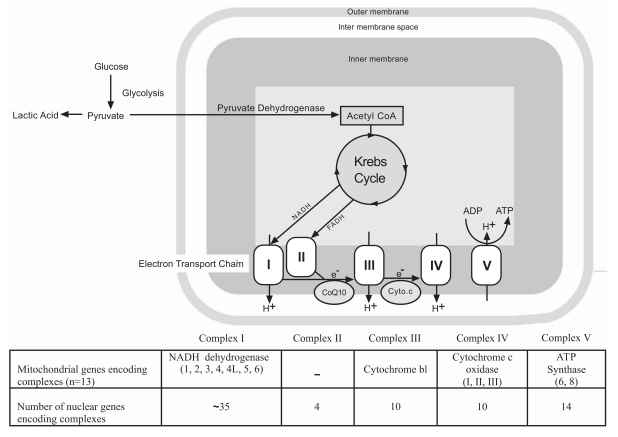
Mitochondrial Structure and Function.

Mitochondria are intracellular organelles in the cytoplasm known as the power houses of the cell. They play a crucial role in adenosine 5’-triphosphate (ATP) production through oxidative phosphorylation (OXPHOS). The latter process is carried out by the electron transport chain made up of Complex I (NADH: Ubiquinone oxidoreductase or dehydrogenase), Complex II (Succinate:Ubiquinone oxidoreductase), Complex III (Coenzyme Q:Cytochrome c reductase or cytochrome bc1 complex), and Complex IV (Cytochrome c oxidase) required to convert food sources to cellular energy. The electron transport system is situated in the inner membrane of the mitochondria and contains proteins encoded by both nuclear and mitochondrial DNA [30]. About 100 proteins coded by both mitochondrial and nuclear genes are required for assembly of the complexes for the respiratory chain [31, 32]. The mitochondrial genome encodes 13 of these 100 proteins [33] (see Fig. **1**).

Human mitochondrial DNA (mtDNA) is a circular double stranded DNA molecule contained within the mitochondrion and inherited solely from the mother. The evolutionary antecedents are bacterial plasmids. Each mitochondrion contains 2-10 mtDNA copies organized into nucleoids [33]. This organization is important to carry out segregation, replication and transcription of mtDNA [33, 34]. In humans, 100-10,000 separate copies of mtDNA are usually present per cell, although an ovum contains about 50,000-5,000,000 mitochondria [33-35] each containing mtDNAs, while sperm cells only have about 50-100 mitochondria each. 

**Fig. (2).**
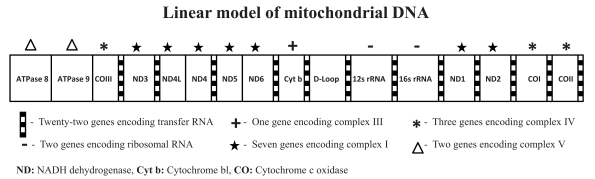
Linear Model of the Mitochondrial DNA Molecule.

Mitochondria are dynamic organelles that fuse and produce fission by changing shapes and size throughout the life of the cell. Thus far, the mitochondria cannot yet be accurately quantified but mtDNA can be analyzed. In mammals, each double-stranded circular mtDNA molecule consists of 15,000-17,000 base pairs containing a total of 37 genes (for production of 13 oxidative phosphorylation proteins used to generate ATP, 22 genes for transfer RNA and two for ribosomal RNA) (see Fig. **2**). When a mutation arises in a cellular mtDNA, it creates a mixed intracellular population of mutant and normal molecules known as heteroplasmy. When a cell divides, it is a matter of chance whether the mutant mtDNAs will be partitioned into one daughter cell or another. Therefore, over time, the percentage of mutant mtDNAs in different cell lineages can drift toward either pure mutant or normal (homoplasmy), a process known as replicative segregation, which then impacts on cellular energy and human diseases [36-38]. 

Mitochondrial disorders may present in early childhood or later in life. Human mitochondrial diseases due to homoplasmy for all mutant mtDNA copies often present early in childhood. Heteroplasmy may involve only a few mutant copies with the remaining copies being unaffected. Individuals with heteroplasmic mutational disorders may not display symptoms until adulthood or after many cell divisions, whereby a threshold number of abnormal mitochondria with mutant alleles result in disease. The disease Neuropathy with muscle weakness, Ataxia, and Retinitis Pigmentosa (NARP) is caused by T8993G heteroplasmic mtDNA mutations. When heteroplasmy is present in less than 10 percent of copies, NARP findings are usually absent. At 10-70 percent, adult onset NARP is present, and at 70-100 percent childhood Leigh syndrome occurs. Other examples of known mitochondrial diseases include Myoclonic Epilepsy with Ragged Red Fibers (MERRF) and the disorder known as Mitochondria Encephalomyopathy, Lactic Acidosis, and Stroke-like episodes (MELAS) (summarized by Wallace [37, 38]). 

In the mitochondria ATP production, free oxygen radicals and reactive oxygen species (ROS) are produced and then normally removed from the cells by anti-oxidant enzymes. When the production of ROS and free radicals exceeds the limit, oxidative stress occurs, that is, ROS combine with lipids, nucleic acids and proteins in the cells leading to cell death by apoptosis or necrosis [39]. Since brain cells have limited antioxidant activity, a high lipid content and high requirement for energy, it is more prone to the effects of oxidative stress [40]. 

Coleman and Blass in [41] were the first to link to bioenergy metabolism disturbances with ASD. They reported lactic acidosis in four children with autism. Later, Laszlo *et al.* [42] reported increased serotonin, lactic acid and pyruvate levels in children with autism. Lombard [43] then proposed that mitochondrial oxidative phosphorylation defects could cause abnormal brain metabolism in children with autism, leading to lactic acidosis and decreased serum carnitine levels. Conversely, Correia *et al.* [44] studied 196 children with autism and found that 17% had high lactic acid levels and 53 had an elevated lactate to pyruvate ratio. Additionally, muscle biopsies performed in 30 of the children with autism showed a mitochondrial defect in 7 of the children. Muscle biopsies studied by Tsao and Mendell [45] in two unrelated girls with autism showed partial complex I deficiency and coenzyme Q10 deficiency in one girl and partial deficiency of complexes II, III and IV in the other. Both girls had normal serum amino acids, ammonia, lactate, urine organic acids and normal karyotyes as well as negative DNA studies for Rett and fragile X syndromes. 

Recently, Shoffner and colleagues [46] studied 28 children diagnosed with ASD for mitochondrial diseases and found high levels of lactate, pyruvate and alanine in the blood, urine and cerebrospinal fluid in 14 of the children. Using muscle biopsies, they identified a mitochondrial complex I defect in 50% (14/28) of the affected children, combined complex I and III defects in 18% (5/28), combined complex I, III and IV defects in 18% (5/28) and isolated complex V defects in 14% (4/28). Seventy-one percent (20/28) of the children had abnormal oxidative phosphorylation after performing protein assessment studies on selected subunits for complexes I, II, III and IV. Their results suggested an even greater link between ASD and impairment in mitochondrial function. (see Table **1** for a summary of known mitochondrial and nuclear gene defects in ASD). 

### Mitochondrial Gene Defects 

Early cases reported by Graf and colleagues [47] provide examples of mtDNA mutations and enzyme defects. They described a brother and sister with mtDNA G8363A transfer RNA (tRNAlys) mutations. The 3 year old boy met the diagnostic criteria for autism at 4 years of age and showed a 60% mutation rate in muscle tissue and 61% in blood. His mitochondrial markers of lactate and pyruvate levels were normal following a carbohydrate loading test. The activity of the mitochondria respiratory chain (MRC) was measured for complexes I, II, III, IV and citrate synthetase. This boy had high activity for mitochondrial enzyme levels suggested by ratios of complex I to citrate synthetase, but normal activity for complexes II, III and IV. His sister at age 6 years was diagnosed with Leigh syndrome, a neurometabolic disorder affecting the brain and manifesting between 3 months and 2 years of age due to mutations of mtDNA or nuclear genes involving mainly complex I. She was found to have the same mtDNA mutation but present at an 86% level in the muscle and 82% in blood, with increased lactate levels and low mitochondrial enzyme activity. This study of G8363A mtDNA mutations in autism stresses the importance of biochemical mitochondrial markers and mtDNA mutations requiring further investigations. 

In addition, Pons *et al.* [48] followed five unrelated children with ASD and reported abnormal respiratory chain enzyme activity. A mtDNA gene mutation at position A3243G was found in four of the five children. High cerebral lactic acid levels were identified using brain magnetic resonance spectroscopy in three of the five subjects. One child with ASD had an older sister with the mtDNA deletion syndrome (MDS), that is, a decreased quantity of mtDNA with a reduced number of mtDNA molecules per cell. MDS is an autosomal recessive disorder characterized by a quantitative defect in the copy number or amount of mtDNA and recently linked to two nuclear genes, *dGK* and *TK2.* The sister had no depletion of the mtDNA or mutations in the two nuclear genes. However, they did have mtDNA depletion in the skeletal muscle at a 72% level. These findings highlight the importance of the A3243G mtDNA mutation in ASD and the investigators proposed that mutations or depletion of mtDNA should be investigated in other children presenting with ASD.

Oliveira *et al.* [49] reported to date the only population-based ascertainment of children with ASD and mitochondrial dysfunction. They identified high lactate levels in 14 of 69 children with ASD. High lactate-to-pyruvate level ratios occurred in 11 of the 14 children; deltoid muscle biopsies were performed in the 11 children for respiratory chain abnormalities. Point mutations were examined in the mtDNA at positions G8363A, A3243G, T3271C, T3256C, and T8356C, as well as mtDNA levels or depletions. While 5 of the 11 children showed definite mitochondrial respiratory disorders, using the clinical and laboratory diagnostic criteria reported by Bernier *et al.* [50], but no mtDNA genetic defects were observed. Later, Oliveira *et al.* [51] reported on genetic and associated medical conditions occurring in children with ASD and showed 5 percent with chromosome disorders, 4 percent with mitochondrial disorders, 3 percent with single gene disorders, 3 percent with infectious diseases, 3 percent with genomic syndromes or birth defects and the remaining cases having idiopathic (unknown) causes. Their study further confirmed the role of the electron transport chain in the mitochondria and autism. Although they did not find large deletions in the mitochondrial DNA, lack of analysis for microdeletions, mutations and depletion of mtDNA were limitations of the study.

An interesting study by Fillano *et al.* [52] further illustrates the importance of the mitochondria in children with features of ASD. They evaluated 12 children with HEADD syndrome (hypotonia, epilepsy, autism and developmental delay) for mitochondrial abnormalities by examining electron transport chain respiratory activity, mitochondrial structure and deletions or point mutations of mtDNA genes or the amount of mtDNA present. They documented decreased activity of the mitochondrial respiratory complexes (I, III, IV and V) mainly in enzymes encoded by mtDNA, in seven of eight subjects studied. However, seven individuals had normal mitochondrial structure while five had decreased or depleted amounts of mtDNA. Lastly, structural defects in the mitochondria were found in three of the four subjects studied. Interestingly, seven children with mitochondrial enzyme defects had no mutations or structural defects in the mitochondria and only one child with a mutation had no enzyme defects. Thus, it is too preliminary to hypothesize that enzyme defects are independent of mtDNA mutations or structural defects. The presence of enzyme defects at a later age is unknown. More studies are required to follow these children into later life. In this study, mtDNA point mutations were not seen although a 7.4kb mtDNA mutation was observed in a child. Later, Pons *et al.* [48] found that 72% of the mtDNA genome was absent or depleted in a child with PDD having a history of mtDNA depletion syndrome in an older sister. The use of newer genetic techniques such as mtDNA microarrays and sequencing along with related nuclear genes (coding and non-coding RNA) will be useful to characterize the mitochondrial genome for depletion status or mutations. 

### Relationship of Nuclear Genes

Studies reviewed by Smith *et al.* [53] linked nuclear genes and mtDNA in ASD, for example, the nuclear gene, *SLC23A12.* This most important gene located on chromosome 21q31 encodes a calcium-binding carrier protein used by the mitochondria. It is involved in the exchange of the amino acid aspartate for glutamate in the inner membrane of the mitochondria for use in the electron transport chain. Ramoz *et al.* [54] identified two single nucleotide polymorphisms (SNPs-rs2056202 and rs2292813) in the *SLC25A12* gene associated with ASD in their study of 411 families. This association was confirmed by Seguarado *et al.* [55] and by Silverman *et al.* [56].

A recent study by Ezugha *et al.* [57] of a 12-year old male with dysmorphic facies, intellectual disability, autism, epilepsy and leg weakness identified decreased activity of mitochondrial complexes I and IV. A 1 Mb deletion was found on chromosome 5q14.3 band. They suggested that the features seen in this child were consistent with mitochondrial dysfunction and a nuclear gene is located on chromosome 5 coding for protein involved in activity of these mitochondrial complexes. Correia *et al.* [44] also studied the linkage of this gene in an additional 241 families and found high lactate levels and lactate/pyruvate ratios. However, they did not find SNPs for the *SLC23A12* gene in their families with autism. Also, Chien *et al.* [58] studied 465 individuals with ASD but did not find any SNP changes in the *SLC23A12* gene.

### Role of the Nuclear Genome in Mitochondrial DNA Replication, Maintenance and Depletion

Mitochondrial DNA replication and maintenance is regulated by enzymes which are proteins encoded by nuclear DNA genes [59]. One such nuclear gene is DNA polymerase gamma 1 *(POLG1*) which codes for mitochondrial DNA polymerase [60]. This gene is involved in the production of mtDNA and has been linked to various mitochondrial conditions. Therefore, mutations in nuclear genes required for mtDNA replication may affect mtDNA function. 

Studies have examined the role of nuclear genes in causing depletion of the mtDNA genome and tissue specific conditions [61, 62]. Mandel and colleagues [61] observed neuronal symptoms with increased lactic acid levels in 19 children with deoxyguanosine kinase *dGUOK* gene mutations and hepatic failure with reductions in activity of mitochondrial complexes I, II and IV. However, the enzyme activity for complex II, which is encoded by nuclear DNA, was normal in these patients. Furthermore, quantitative mtDNA analysis showed a reduced mtDNA/nuclear DNA ratio of 8-39% in liver specimens from seven of the children. 

The main supply for dNTPs required for energy comes from the deoxynucleoside salvage pathway regulated by two mitochondrial enzymes *dGK* and *TK2,* encoded by the nuclear genome [63, 64]. Hence, Mandel *et al.* [61] examined nuclear genes and found that *dGK* was mutated in patients with reduced mtDNA/nuclear DNA ratios. A similar study by Saada *et al.* [62] in two of four individuals with known mitochondrial depleted myopathy showed a mutation in the *TK2* nuclear gene. These two studies further supported that nuclear genes are linked to mtDNA but more research is needed to determine their role in autism and for identification of mtDNA genetic defects, mutations or for the mtDNA depletion status associated with related nuclear genome defects.

Polymerase gamma (*POLG1*) is an important polymerase enzyme responsible for mtDNA replication and base excision repair [60]. The nuclear gene encoding the *POLG1 *enzyme is located on chromosome band 15q25 [65, 66]. Any mutation in this gene will impact mtDNA replication and repair processes. Interestingly, in humans, maternal disomy 15 (both chromosome 15s from the mother) is seen in about 25% of subjects with Prader-Willi syndrome (PWS), the most common known syndromic cause of morbid obesity in humans [67]. PWS is frequently associated with ASD particularly in those subjects with PWS due to maternal disomy 15 and associated with a lower metabolic rate [68]. Therefore, maternal disomy 15 in PWS could also influence the activity of the *POLG1* gene. Studies have shown that mutations or disturbances of the *POLG1* gene cause deletions of mtDNA genes. Additionally, autism is linked to defects of human chromosome 15q11-q13 region including inverted duplications found in about 5% of cases with autism [12]. Filipek *et al.* [69] further reported that subjects with 15q11-q13 duplications and ASD have increased mitochondria and mitochondrial respiratory chain blockage at complex IV, but lactate levels were not significantly elevated. While this study did not examine for mtDNA mutations, it established a link between genes in the nucleus specifically on chromosome 15q11-q13 and an interplay with mitochondrial function in subjects with autism. 

Van Goethem *et al.* [70] showed that autosomal dominant progressive external opthalmoplegia (adPEO) is due to *POLG1* gene mutations leading to mtDNA deletions. Apart from *POLG1*, the autosomal dominant type of PEO has been linked to the *TWINKLE* gene on chromosome 10q [35] and the *ANT1* gene on chromosomal 4q [71]. These nuclear gene mutations are known to cause mtDNA disturbances.

*POLG1* has been linked to many conditions apart from adPEO. For example, it causes autosomal recessive PEO, sensory-ataxic neuropathy, dysarthria and opthalmoplegia (SANDO), juvenile spino-cerebellar ataxia-epilepsy syndrome (SCAE) and Alpers-Huttenlocher hepatopathic poliodystrophy. These encephalomyopathies are caused by abnormal nuclear mitochondrial intergenomic cross-talk linked to *POLG*1 gene mutations and to mtDNA deletions. This strong link between nuclear genes and the mtDNA genome is further supported by a prevalence of 2% of individuals in the general population carrying this nuclear gene defect [59]. 

Another nuclear gene that can cause multiple mtDNA abnormalities including duplications, deletions and point mutations is thymidine phosphorylase (TP) [72, 73]. The TP nuclear gene is located on chromosome 22q13 [74] and encodes the cytosolic enzyme regulating pyrimidine nucleoside levels through phosphorolytic thymidine catabolism to thymidine and ribose [75]. Mitochondria neurogastrointestinal encephalopathy (MNGIE) results from loss of TP activity. Hence, the above studies have shown that mutations of nuclear genes *TK2, DGK, PLOG, TWINKLE, ANT1* and *TP* can cause deletions and depletions of mtDNA, their involvement and frequency in ASD requires further investigation.

Finally, oxidative stress and gene methylation (epigenetics) are both considered to play a role in producing ASD. Oxidative stress in brain cells due to genetic or environmental causes, leads to decreased methionine synthase activity [76]. Methionine synthase action involves two processes: a) dopamine-stimulated phospholipid methylation which is required for synchronizing normal brain activity during attention; and b) DNA methylation which silences gene activity. During DNA methylation a methyl group is added to the cytosine-phosphate-guanine (CpG) site and the methylated cytosine is converted to 5-methylcytosine; thus, inactivating gene expression. This is a normal process. One can propose that the important interaction between methionine metabolism and methylation with overall reactions of folate and B12 metabolism is regulated by mitochondrial reduction/oxidative reactions controlling nuclear gene methylation (epigenetics or methylomics) and glutathione metabolism. When disturbed, this process contributes to ASD [77]. When methionine synthase activity is impaired, children may manifest attentional impairment and other symptoms from defective gene expression leading to ASD. 

Deth *et al.* [78] further reported the involvement of various genes in the process of methylation with the most important being methyl-CpG-binding (MeCP2) protein. Abnormal MeCP2 protein is linked to Rett syndrome and ASD [79]. MeCP2 participates in gene silencing by binding to the methylated genes in the brain. Any mutation of this gene impacts on the expression status of known methylated genes and therefore interferes with epigenetics. 

Oxidative stress is also known to cause deletions of the mtDNA genes in mice models [80]. For example, chronically-administered intraperitoneal injections of the chemotherapeutic agent, doxorubicin, in mice has resulted in a 4kb deletion of the mtDNA in heart muscle. In addition, Lu *et al.* [81] indirectly confirmed these findings by reporting mtDNA deletions in ageing skin from humans with increased free oxygen radicals and reactive oxygen species and further support the basis for examining mtDNA or gene deletions in ASD.	 

To further evaluate for mitochondrial dysfunction and mtDNA abnormalities, Giulivi *et al.* [82] recently studied lymphocytes from 10 children with ASD and 10 controls aged 2 to 5 years. The outcome measures included oxidative phosphorylation capacity at the cell level, mtDNA copy number, mitochondrial rate of hydrogen peroxide production, and plasma lactate and pyruvate levels. The majority of the children with ASD (6 of 10) had lower mitochondrial complex I activity than found in controls. Higher plasma pyruvate levels occurred in 8 of 10 children with ASD compared with controls consistent with a lower pyruvate dehydrogenase activity. Hence, children with ASD had higher mitochondrial rates of hydrogen peroxide production than in controls. Mitochondrial genome studies showed mitochondrial DNA overreplication in 5 children with ASD and mtDNA deletions involving the segment coding for cytochrome b in 2 children with ASD. Therefore, the children with ASD were more likely to have mitochondrial dysfunction, mtDNA overreplication, and/or mtDNA deletions than typically developing children.

In summary, the role of the mitochondria and mitochondrial defects are discussed in relationship to ASD. Several studies have linked autism to defects in oxidative phosphorylation encoded by mtDNA along with interaction of nuclear genes. Individuals with the ASD phenotype clearly show genetic-based primary mitochondrial disease. Further studies with the latest genetic technology such as next generation sequencing, microarrays, bioinformatics and biochemical assays will be required to determine the prevalence and type of mitochondrial defects in ASD. Elucidation of molecular abnormalities resulting from mitochondrial or genetic defects in individuals with ASD may lead to better treatment options and outcomes.

## Figures and Tables

**Table 1.** Reports of Autism Spectrum Disorders (ASD) Involving Mitochondrial and Nuclear Gene Defects

References	Number of Subjects with ASD	Sample	MitochondrialComplex Defects	MitochondrialGene Defects	Nuclear Gene Defects	Other
Laszlo *et al*., 1994	30	Blood			Chromosome 18q deletion(1 of 30 subjects)	LA ↑ (13 of 30 subjects);Pyruvate ↑ (9 of 30 subjects)
Graf *et al*., 2000	1	Blood, Muscle	I, IV	tRNALys(G8363A)		
Fillano *et al*., 2002	12	Muscle	I, III, IV, V (7 of 8 subjects)	mtDNA deletion (5 of 12 subjects);mtDNA defect (3 of 12 subjects)		
Filipek *et al*., 2003	2	Muscle	III		Chromosome 15q11-q13 duplication(2 of 2 subjects)	Carnitine ↓(2 of 2 subjects);Plasma alanine ↑ (1 of 2 subjects);Lactate ↑ (1 of 2 subjects);CPK ↑ (1 of 2 subjects)
Pons *et al*., 2004	5	Muscle	I, II, III, IV	tRNALeu (A3243G)(2 of 5 subjects)		Plasma lactate ↑ (2 of 3 subjects);CSF lactate ↑ (1 of 1 subject)
Ramoz *et al*., 2004	411	Blood			SNP (rs2056202 and rs2292813)in *SLC25A12* of chromosome 2q31(197 of 411 subjects)	
Oliveira *et al*., 2005	69	Muscle	I, IV, V	No mutations seen		Plasma lactate ↑ (14 of 69 subjects)
Segurado *et al*., 2005	174	Blood,Buccal cells			SNP (rs2056202 and rs2292813)in *SLC25A12* (158 of 174 subjects)	
Correia *et al*., 2006	210	Muscle	Multiple complex defects		Normal *SLC25A12*	LA ↑ (36 of 210 subjects)
Tsao & Mendell, 2007	2	Muscle	II, III, IV		SNP (rs2056202 ) in *SLC25A12*	CoQ10 ↑ deficiency (1 of 2 subjects)
Weissman *et al*., 2008	25	Blood, Muscle	I (16 of 23 subjects),II (2 of 23 subjects), III (5 of 23 subjects),IV (1 of 23 subjects)	mtDNA deletion(7 of 25 subjects)		Plasma lactate ↑ (19 of 25 subjects); Plasma pyruvate ↑ (9 of 17 subjects); Plasma CPK ↑ (8 of 25 subjects)
Silverman *et al*., 2008	355	Blood			SNP (rs2056202 and rs2292813)in *SLC25A12* (170 of 355 subjects)	
Shoffner *et al*., 2010	28	Muscle, BloodCSF, Urine	I (14 of 28 subjects);I+III (5 of 28 subjects); V (4 of 28 subjects);I+III+IV (20 of 28 subjects)	mtDNA deletion(1 of 20 subjects)		Plasma lactate ↑;CoQ10 deficiency (1 of 14 subjects)
Giulivi *et al*., 2010	10	Blood	Decreased NADH oxidase (8 of 10 subjects);I (6 of 10 subjects);IV (3 of 10 subjects);V (4 of 10 subjects)	mtDNA over-replication (5 of 10 subjects);Cytochrome b1 gene deletion (2 of 10 subjects)		Succinase oxidase (6 of 10 subjects);Pyruvate dehyrogenase (8 of 10 subjects)
Ezugha *et al*., 2010	1		I, IV		1Mb deletion in chromosome 5q14.3	

CPK = creatine phosphokinase; LA = lactic acidosis; CSF = cerebral spinal fluid.
